# Genetic Control Diversity Drives Differences Between Cadmium Distribution and Tolerance in Rice

**DOI:** 10.3389/fpls.2021.638095

**Published:** 2021-02-19

**Authors:** Yi-Bo Chen, Yu-Chao Chen, Yu-Xing Zhu, Sai Li, Hua-bing Deng, Jiu-Rong Wang, Wen-Bang Tang, Liang Sun

**Affiliations:** ^1^College of Agronomy, Hunan Agricultural University, Changsha, China; ^2^Key Laboratory of Agro-Ecological Processes in Subtropical Region, Institute of Subtropical Agriculture, Chinese Academy of Sciences, Changsha, China

**Keywords:** rice, Cd distribution, Cd tolerance, QTL, genetic control diversity

## Abstract

Rice, a staple crop for nearly half the planet’s population, tends to absorb and accumulate excessive cadmium (Cd) when grown in Cd-contaminated fields. Low levels of Cd can degrade the quality of rice grains, while high levels can inhibit the growth of rice plants. There is genotypic diversity in Cd distribution and Cd tolerance in different rice varieties, but their underlying genetic mechanisms are far from elucidated, which hinders genetic improvements. In this study, a joint study of phenotypic investigation with quantitative trait loci (QTLs) analyses of genetic patterns of Cd distribution and Cd tolerance was performed using a biparent population derived from *japonica* and *indica* rice varieties. We identified multiple QTLs for each trait and revealed that additive effects from various loci drive the inheritance of Cd distribution, while epistatic effects between various loci contribute to differences in Cd tolerance. One pleiotropic locus, *qCddis8*, was found to affect the Cd distribution from both roots to shoots and from leaf sheaths to leaf blades. The results expand our understanding of the diversity of genetic control over Cd distribution and Cd tolerance in rice. The findings provide information on potential QTLs for genetic improvement of Cd distribution in rice varieties.

## Introduction

Cadmium (Cd), a heavy metal element that is nonessential and toxic to most organisms, is ubiquitously distributed in soil as a consequence of anthropogenic activities, where it can bring serious damage to crop production (Zhao et al., 2015). Exposure to high Cd concentrations have negative effects on photosynthesis, essential element uptake, and the stability of gene expression and cause oxidative stress (Benavides et al., 2005). Furthermore, the growth and development of crop plants can be substantially suppressed, resulting in morphological aberrations or fertility degradation and ultimately losses in biomass and yield (Dalcorso et al., 2008). Exposure to low levels does not tend to disrupt growth and development, but uptake of essential elements, such as Zn, Fe, and Mn, may be hindered by uptake of Cd, and Cd can gradually accumulate in the edible parts, reaching high levels (Benavides et al., 2005; Clemens, 2006; Yamaji and Ma, 2014; Li et al., 2017). This results in changes in physiological and biochemical components followed by a reduction in crop quality, representing a threat to food safety and human health (Clemens et al., 2013; Zhao et al., 2015).

Rice possesses some resistance to Cd and also prefers to accumulate Cd in grain to high levels, and this is a major challenge for areas producing and consuming rice as a staple food, especially in Asian countries (Clemens et al., 2013; Zhao et al., 2015; Sun et al., 2016). Based on the physiological processes of Cd uptake, transport, and accumulation in rice, a “phloem-tropic” mode has been proposed involving preferential translocation of Cd through the phloem (Yamaji and Ma, 2014). *In vitro*, Cd is first absorbed in rice roots then released into the xylem. After xylem-to-phloem loading and detoxification processes, including sequestering in vacuoles and binding to cell wall compounds, absorbed Cd is distributed through vascular bundles (VBs) in nodes, which are interconnected and linked with roots, stems, sheathes, leaves, and panicles (Clemens and Ma, 2016; Tan L. T. et al., 2020). Through the VB system, Cd is preferentially distributed to developing tissues, and it eventually accumulates in rice grains (Yamaguchi et al., 2012; Yamaji and Ma, 2014). Numerous studies have been carried out to explore the molecular mechanisms of these processes in rice, and a series of genes and quantitative trait loci (QTLs) associated with Cd uptake and distribution have been characterized, including *OsCd1*, *OsNramp1*, *OsNramp5*, *OsHMA2*, *OsZIP7*, *OsZIP5*, *OsZIP9*, *CAL1*, and *OsHMA3* (Ueno et al., 2010; Takahashi et al., 2011; Sasaki et al., 2012; Yamaji et al., 2013; Luo et al., 2018; Tan et al., 2019; Yan et al., 2019; Tan L. T. et al., 2020).

After uptake into roots, Cd is translocated into different rice tissues, where it causes local and systemic toxicity, including growth inhibition, photosynthesis damage, metal-induced oxidative damage, and alternation of metabolic enzyme activity (Clemens, 2006). In response to Cd toxicity, rice have evolved various physiological processes. For example, certain classes of compounds are secreted, such as organic acids, peptides, and polysaccharides, which may also participate in sequestering Cd to alleviate Cd toxicity (Clemens, 2006; Luo et al., 2018). At the molecular level, a number of genes are directly or indirectly associated with Cd tolerance, including *OsCDT1*, *DEP1*, *OsCLT1*, *miR268*, and *OsCADT1* (Kuramata et al., 2009; Kunihiro et al., 2013; Ding et al., 2017; Chen et al., 2020). Complex biological mechanisms have been found to mediate Cd tolerance, mostly related to Cd uptake, transport, translocation, compartmentalization, and sequestration (Clemens and Ma, 2016). During uptake and translocation, Cd can be sequestrated in the cell wall and vacuole (Zhang et al., 2009). Excess Cd can also be compartmentalized into less Cd-sensitive organs. For instance, to avoid inhibition in developing tissues, such as shoots in seedlings, Cd is sequestered in rice roots to decrease Cd transported upwards into the rest of the plant (Sasaki et al., 2014).

There is genotypic variation associated with Cd distribution and Cd tolerance among different rice varieties (Uraguchi and Fujiwara, 2012; Pinson et al., 2015; Tan Y. J. et al., 2020), and a series of associated QTLs have been identified and utilized for genetic improvements (Kashiwagi et al., 2009; Xue et al., 2009; Zhang et al., 2014; Liu X. et al., 2019; Lu et al., 2019; Zhou et al., 2019; Tan Y. J. et al., 2020; Wang et al., 2020). However, in consequence of lacking joint studies on genetic patterns of Cd distribution and Cd tolerance, especially regarding allelic differences, the genetic relationships between these traits remain being poorly understood. Thus, exploring the genetic variation responsible for simultaneous Cd distribution and Cd tolerance is important to support the development of lower grain Cd accumulation in rice through genetic improvement. To this end, we herein implemented a joint study of phenotypic investigation with QTL analysis for genetic patterns and assessed potential QTLs related to Cd distribution and Cd tolerance. The results could broaden our understanding of the genetic basis driving these traits in different rice varieties, and the findings may prove helpful for exploring functional alleles that could be utilized in low Cd rice breeding programs.

## Materials and Methods

### Plant Materials and Growing Conditions

To investigate the genetic pattern relationships between Cd distribution and Cd tolerance, two rice varieties and their derived bipopulation were employed: the typical *indica* rice variety “93-11” and *japonica* rice variety “IRAT129,” high and low Cd accumulation varieties, respectively (Sun et al., 2016; Zhou et al., 2019). The biparent population of recombinant inbred lines (RILs) derived from intercrossing the two varieties was generated using a single-seed descent approach. The F_1_ hybrid was planted for successive selfing to generate the genetic population. Finally, the F_10_ RIL population consisting of 147 lines was harvested for physiological and genetic studies.

To investigate the differences in Cd distribution and Cd tolerance, physiological assays were carried out on RILs using hydroponic system with 1/2 Kimura B solution (KB) made from a 20× KB nutrient stock solution prepared as described previously (Tan et al., 2019, Tan L. T. et al., 2020). After pregermination at 28°C for 4 days, germinated seeds of RILs and their parental varieties were transplanted (six biological replicates) and precultured in 3 L of 1/2 KB solution for 3 weeks in a temperature-controlled greenhouse (28°C 12 h day/22°C 12 h night). All physiological assays included two biological replicates. For Cd treatments, seedlings of RILs were transplanted into nutrient solution containing 2.5 μM Cd (CdSO_4_, pH 5.4) for analysis of Cd distribution (two biological replicates) or 25 μM Cd (CdSO_4_, pH 5.4) for analysis of Cd tolerance (two biological replicates). Additionally, two biological replicates were simultaneously cultured without Cd (pH 5.4) as controls in Cd tolerance experiments. Cd exposure treatments lasted 1 week, 1/2 KB solution was renewed every 2 days, and the pH was maintained at pH 5.4 using 1 M KOH solution.

### Building the Linkage Genetic Map

Each line of the 147 RILs was genotyped to build a genetic linkage map. DNA extractions were implemented using the classical cetyltrimethyl ammonium bromide (CTAB) method with minor modifications (Sun et al., 2016). A total of 237 genomic sequence tagged site (STS) markers, based on STS polymorphisms between “93-11” and “NPB” varieties in public databases^1^, were screened for available markers, displayed as distinct PCR product polymorphisms between “93-11” and “IRAT129.” All STS markers were developed and labeled in a previous study (Li and Mao, 2018). Meanwhile, another 71 simple sequence repeats (SSRs) primers, based on the GRAMENE database^2^, were used to survey marker polymorphisms to fill gaps in the genetic map (Supplementary Data 1). The SSR assay was performed as described previously (Sun et al., 2016). A total of 148 markers, including 107 STS markers and 41 SSR makers, were used to genotype each line of RILs. The linkage map consisting of 148 markers was generated using the Mapmaker/Exp 3.0 program (Lincoln et al., 1992). The graphical genotypes of the 148 polymorphic markers in the 147 RILs were identified and visualized using Icimapping version 4.0 (Li et al., 2007). In general, the 148-marker genetic map covered all 12 chromosomes. The genetic length of the map was estimated as 1793.7 cM with an average distance of 12.2 cM between pairs of markers (Supplementary Figure 1).

### Determination of Cd Distribution and Cd Tolerance

All tested individuals, including the two parental lines and the 147 RIL lines, were harvested after culturing in the presence of Cd in hydroponic assays. After culturing under 2.5 μM CdSO_4_ conditions, 1-month-old seedlings were soaked in a solution of 5 mM CaCl_2_ for 15 min to remove adhered Cd, then rinsed three times with distilled water. To investigate the Cd distribution patterns, seedlings were partitioned into root, basal internode (node, Nd), leaf sheath (Ls), and leaf blade (Lb) samples. All tissue samples, including roots, nodes, and leaves (as Lf, including leaf sheaths and leaf blades) were then dried in an oven at 80°C to constant weight, and ∼0.3000 g of each sample was digested by concentrated nitric acid (100%) in a microwave digestion system (Milestone ETHOS UP, Italy). The Cd level of each digested solution was determined by inductively coupled plasma optical emission spectrometry (ICP-OES) using an Agilent 700 Series instrument (Agilent Technologies, United States). Blank and quality controls (Certified Plant Reference Material, GBW10045) were included in parallel to confirm the accuracy of Cd determinations.

For Cd tolerance, plants simultaneously cultured in both 0 and 25 μM CdSO_4_ hydroponic assays were harvested. To investigate the phenotypic variation in Cd tolerance, the lengths of shoots and roots were measured after removing adhered Cd. Shoot length was measured from the basal internode to the tip of the longest leaf, root length was measured from the basal internode to the tip of the longest root, and five to eight individuals from each line were surveyed. Growth conditions for each seedling were defined as the growing ratio calculated as the growth ratio dividing the length of shoots to that of the roots. The relative growing ratios between control (0 μM CdSO_4_) and Cd exposure (2.5 μM CdSO_4_) groups were estimated to assess Cd tolerance in both parental lines and the lines from the RIL population.

### QTL Identification and Statistical Analysis

In this study, we used normalization of phenotypic variation to enhance genetic factors to further investigate the variations in Cd distribution and Cd tolerance. Four indicators were developed. For Cd distribution, the Cd concentration ratio between shoots (leaves and nodes) and roots (as S/R), the Cd concentration ratio between developing tissues (leaf sheaths and leaf blades) to nodes (as Lf/Nd), and the Cd concentration ratio between leaf blades and leaf sheaths (as Lb/Ls) were calculated. S/R was used to describe Cd translocation from roots to shoots, Lf/Nd was used to describe Cd transportation from nodes to developing tissues, and Lb/Ls was used to describe Cd distribution between developing tissues. For Cd tolerance, the relative growth ratio between 0 and 25 μM CdSO_4_ (Cd-Tol) was calculated to describe Cd tolerance differences in the RILs.

QTL identification was performed using the average performance of each trait from two biological replicates under an inclusive composite interval mapping (ICIM) approach with the QTL Icimapping 4.0 program, including additive QTLs and epistatic QTLs (Wang, 2009). ICIM was run with a 1-cM window, and the largest *P* value for the stepwise regression-based likelihood ratio test was set at 0.05 for additive QTLs and 0.01 for epistatic QTLs. To avoid false-positive detection and ensure the reliability of QTL analysis, chromosomal regions containing marker loci with a logarithm of odds (LODs) score ≥2.50 were considered significant additive QTLs, and to declare a significant epistatic QTL, the threshold for the LOD score was set at 5.0. Other statistical analyses, such as linear regression and Student’s *t*-tests, were performed mainly with Microsoft Excel 2016 (Microsoft Corporation) and SPSS 18.0 (SPSS Inc. PASW Statistics for Windows).

## Results

### Cd Distribution Pattern Differences Between “93-11” and “IRAT129” Varieties

**FIGURE 1:**
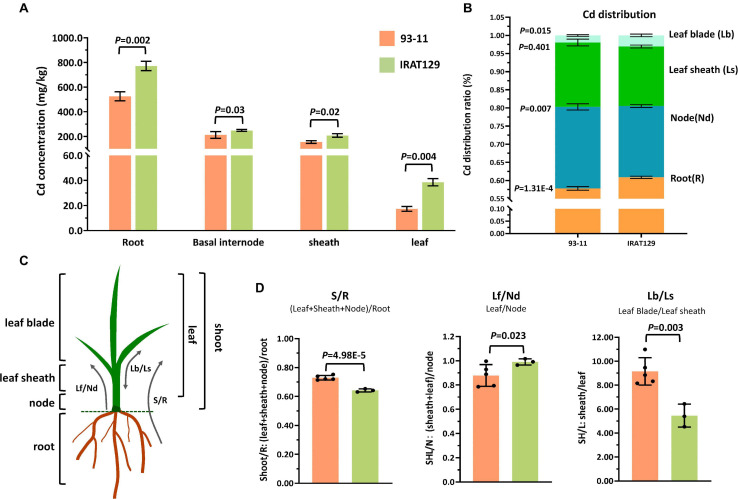
Cd distribution differences between “IRAT129” and “93-11” rice varieties. **(A)** Comparison of Cd levels in roots, basal internodes (nodes), leaf sheaths, and leaf blades between “IRAT129” and “93-11;” **(B)** comparison of the proportion of Cd in tissues (roots, nodes, leaf sheaths, and leaf blades) relative to total Cd in plants; **(C)** schematic view of the four tissues and the three indicators used to investigate the Cd distribution pattern; **(D)** comparison of the three indicators S/R, Lf/Nd, and Lb/Ls between the two varieties.

In our previous study, it was found that rice variety “IRAT129” from *japonica* rice subspecies accumulated low levels of grain-Cd, while “93-11” from *indica* rice subspecies accumulated high levels of Cd in grains (Tan Y. J. et al., 2020). We carried out a hydroponic culture experiment to compare their distribution patterns, and the results of Cd level comparison revealed that, regardless of the Cd levels in “IRAT129” and “93-11,” Cd levels in the four tissues were ordered root > node > leaf sheath > leaf blade (Figure 1A). Cd levels in roots, nodes, leaf sheaths, and leaf blades of “IRAT129” were, respectively, 1. 5-, 1. 2-, 1. 3-, and 2.2-fold of those in “93-11” under 2.5 μM hydroponic conditions (Figure 1A).

The proportions of Cd in tissues relative to total Cd in plants were also estimated to explore Cd distribution differences between the two parental varieties (Figure 1B). Cd distributed to roots and leaf blades accounted for 57.8 and 1.4% of total Cd in “93-11,” respectively, significantly lower than in “IRAT129” (60.9 and 3.1%, respectively; *P* = 1.31E-4; 0.015). By contrast, the value for Cd in the nodes of “93-11” was 22.4%, significantly higher than in “IRAT129” (19.6%; *P* = 0.007). Cd in leaf-sheath tissues of “93-11” and “IRAT129,” respectively, accounted for 17.7 and 16.4% of total Cd in plants, which was not significantly different (*P* = 0.401). These results indicate that more Cd remained in the roots and leaf blades in “IRAT129” than in “93-11.”

Biologically, the four tested tissues play different roles in Cd distribution processes. Roots are responsible for the absorption of Cd, while nodes are central tissues for Cd xylem-to-phloem translocation from roots to shoots and the other tissues, including leaf sheaths and leaf blades (Yamaji and Ma, 2014). Thus, to better describe the Cd distribution pattern differences, three indicators were assessed (S/R, Lf/Nd, and Lb/Ls; Figure 1C). S/R was used to describe the proportion of Cd upwardly transported from the roots, Lf/Nd describes Cd transport from the node to leaf tissues, and Lb/Ls describes the distribution of Cd between leaf blades and leaf sheaths (Figure 1C). Comparisons of the three indicators revealed that S/R and Lb/Ls in “93-11” were significantly higher than in “IRAT129” (*P* = 4.98E-5; *P* = 0.003), while Lf/Nd was significantly higher in “IRAT129” than in “93-11” (*P* = 0.023; Figure 1D). The results revealed differences in Cd distribution patterns in the two varieties under different scenarios. Compared with “93-11,” “IRAT129” sequestrated more Cd in roots, retained less Cd in nodes, and translocated more Cd to leaf tissues, but distributed less Cd to leaf sheaths than to leaf blades.

### Differences in Cd Tolerance Between “93-11” and “IRAT129”

**FIGURE 2:**
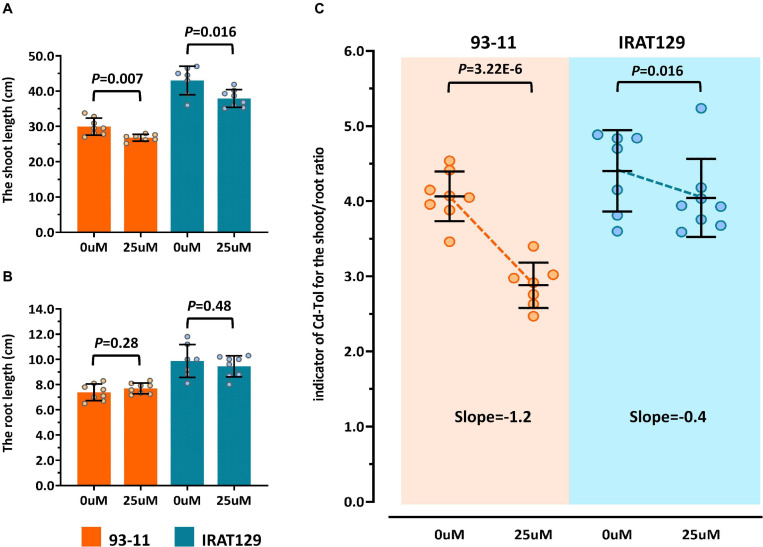
Cd tolerance differences between “IRAT129” and “93-11” rice varieties. **(A)** Comparison of shoot length under 0 and 25 μM Cd stress; **(B)** comparison of root length under 0 and 25 μM Cd stress; **(C)** comparison of Cd-Tol based on changes in the curves of relative growth ratios in “IRAT129” and “93-11.”

According to previous studies (Xue et al., 2009; Huang et al., 2018), the most visible trait of Cd tolerance in rice seedlings is changes in morphology, including changes in the length of roots and shoots. In our preliminary studies, “IRAT129” could grow better than “93-11” under Cd stress (5 μM), suggesting that “IRAT129” is more Cd tolerant than “93-11” (data not shown). In the present study, changes in the length of shoots and roots between controls and 25 μM Cd-stressed plants were quantified to evaluate differences in tolerance between the two rice varieties. Compared with controls, a significant reduction in shoot length was observed under Cd stress; “93-11” exhibited a shoot length reduction of 10.4% compared with a reduction of 11.9% for “IRAT129” (Figure 2A). However, there were no significant differences in root length between the two varieties (*P* > 0.05; Figure 2B).

To explore the changes in morphology in more detail, similar to previous studies (Xue et al., 2009; Huang et al., 2018), changes in shoot/root length between controls and Cd-stressed plants (Cd-Tol) were measured to probe the growth changes following Cd stress. The average Cd-Tol value for “93-11” was 2.88 under Cd stress compared with 4.06 for controls, representing a significant reduction (*P* = 3.22E-6). The average Cd-Tol value for “IRAT129” was 4.04 under Cd stress compared with 4.40 for controls, indicating no significant difference (*P* = 0.106). Reduction curves were subsequently plotted (Figure 2C), and the slope of Cd-Tol variation for “93-11” was 1.2, compared with 0.4 for “IRAT129;” hence, the curve for “93-11” was much steeper than that for “IRAT129” (Figure 2C). Together, these results suggest that “IRAT129” was more tolerant to Cd stress than “93-11.”

### Phenotypic Variation in Cd Distribution and Cd Tolerance in the RIL Population

Based on the observed phenotypic variation of Cd distribution and Cd tolerance, “IRAT129” displayed much greater tolerance to Cd stress and displayed lower Cd distribution from roots to shoots and from leaf sheaths to leaf blades than did “93-11” but higher Cd translocation from nodes to leaves (Figures 1D, 2C). Thus, to investigate the genetic relationship between Cd distribution and Cd tolerance, an RIL population derived from the two varieties was developed to investigate phenotypic variation in the related indicators.

**FIGURE 3:**
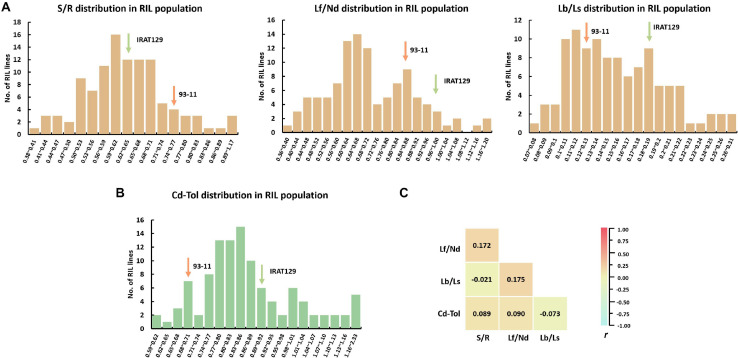
Cd distribution and Cd tolerance performance in the recombinant inbred line (RIL) population derived from “IRAT129” and “93-11” rice varieties. **(A)** Phenotypic distribution of S/R, Lf/Nd, and Lb/Ls in the RIL population; **(B)** phenotypic distribution of Cd-Tol in the RIL population; **(C)** correlation coefficient (*r*) analysis for indicators of Cd distribution and Cd tolerance.

There was great variation for all phenotypic indicators in the RIL population (Figure 3 and Supplementary Table 1). Except for Lf/Nd, the performance of the other Cd distribution and Cd tolerance indicators varied between the two parental varieties (Figures 3A,B). Among Cd distribution indicators, S/R ranged from 0.38 to 1.18 with an average of 0.64, Lf/Nd ranged from 0.37 to 1.39 with an average of 0.72, and Lb/Ls ranged from 0.07 to 0.32 with an average of 0.72. Regarding Cd tolerance, Cd-Tol ranged from 0.61 to 2.33 with an average of 0.88. The coefficient of variation (CV) for the four Cd distribution and Cd tolerance indicators was high (18.6% for S/R, 25.4% for Lf/Nd, 29.6% for Lb/Ls, and 22.5% for Cd-Tol; Supplementary Table 1). Among these indicators, S/R had the lowest CV, suggesting a less variation in Cd transporting from roots to shoots. Thus, the high CV and continuous segregation distributions for all indicators imply that polygenic loci control the phenotypic variation in the RIL population (Figures 3A,B).

To investigate the relationships between Cd distribution and Cd tolerance features, correlation analyses were implemented (Figure 3C). No significant correlations were detected between any of the indicators for Cd distribution; the correlation coefficient (*r*) ranged from −0.021 to 0.175 (*P* > 0.05). These results suggest that the genetic basis for determining the Cd distribution between different tissues differs. Meanwhile, no significant correlation was detected between Cd-Tol and any of the Cd distribution indicators (*P* > 0.05; Figure 3C). These results suggest that the variation in Cd distribution pattern does not endow differences in Cd tolerance, and the genetic pattern of Cd distribution might differ from that of Cd tolerance in our “IRAT129” and “93-11” genetic population.

### Genetic Control Diversity Drives Differences Between Cd Distribution and Cd Tolerance According to QTL Analyses

**FIGURE 4:**
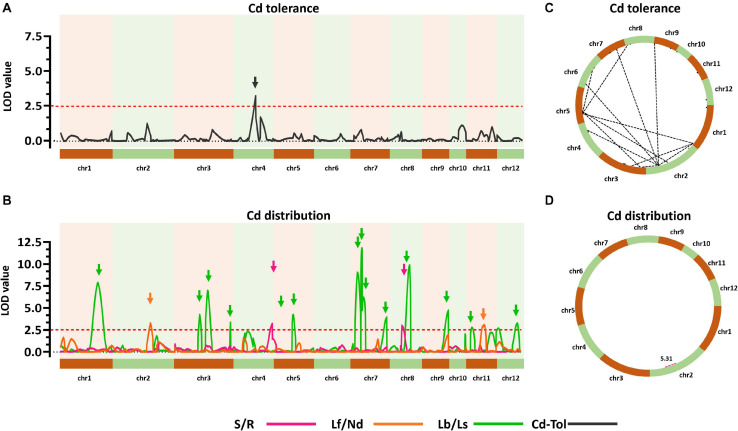
Quantitative trait locus (QTL) analysis of Cd distribution and Cd tolerance variation in the quantitative trait loci (QTL) population. **(A,B)** Additive QTLs for Cd distribution and Cd tolerance. The horizontal axis shows the 12 chromosomes assembled in order. Curves were plotted according to the logarithm of odds (LOD) value calculated with a 5-cM window. Arrows represent QTLs associated with related phenotypic variation in the recombinant inbred lines (RILs). Red dotted lines indicate the threshold of LOD = 2.5. **(C,D)** Epistatic networks of interactions between loci affecting variation in Cd distribution and Cd tolerance in the RIL population. Dotted lines indicate interactions with LOD ≥5.0.

**FIGURE 5:**
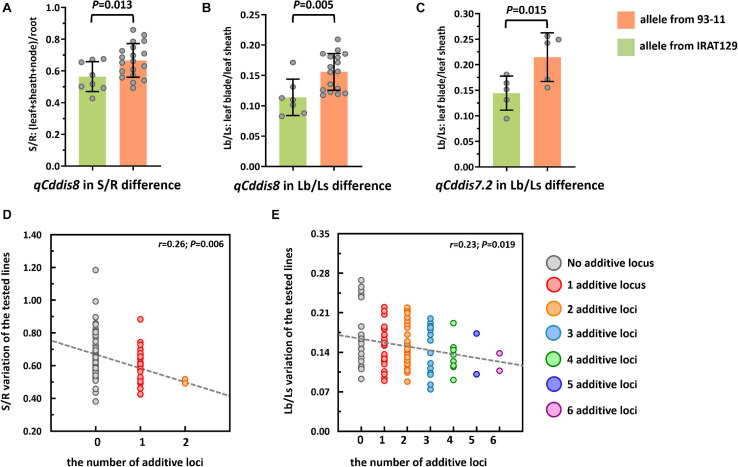
Cd distribution performance of *qCddis8* in the recombinant inbred lines (RILs) and linear regression analysis between Cd distribution variation and the number of additive loci exerting negative effects on phenotypes. **(A,B)** Comparison of S/R and Lb/Ls between the *qCddis8* alleles from “IRAT129” and “93-11;” **(C)** comparison of Lb/Ls between the *qCddis7.2* allele from “IRAT129” and “93-11;” **(D,E)** reduction of S/R and Lb/Ls along with increasing number of additive loci derived from “IRAT129.” No additive locus means that alleles for S/R and Lb/Ls were from “93-11.” Gray dotted lines represent linear regression between the average value of these traits and the number of additive loci. *r* is the correlation coefficient of linear regression.

To explore the genetic controls driving Cd distribution and Cd tolerance, QTL mapping approaches were performed for additive QTLs and epistatic QTLs using average values for four Cd distribution indicators and one Cd tolerance indicator from the RILs. The additive QTL mapping approach revealed a total of 19 additive QTLs explaining the variation in Cd distribution and one additive QTL for Cd tolerance (Table 1). Additive QTLs were detected on all chromosomes except 6 and 10 (Table 1 and Figures 4A,B). Among the detected QTLs, one QTL on chromosome 8 (*qCddis8*) was identified as pleiotropic. This QTL could explain 9.01% of the S/R variation in the RILs with a negative effect of −0.041, and this locus could also explain 7.20% of the Lb/Ls variation in the RILs with a negative effect of −0.019. According to the phenotypic variation, the *qCddis8* allele from “IRAT129” could significantly decrease Cd transport from roots to shoots (*P* = 0.013; Figure 5A), and this allele could also distribute more Cd to leaf sheaths than to leaf blades (*P* = 0.005; Figure 5B). *qCddis7.2*, an additive QTL on chromosome 7, had the highest LOD value (11.70) and could explain 8.62% of Lb/Ls variations in the RILs with a negative effect of -0.021. According to the phenotypic variation after discounting other loci, the *qCddis7.2* allele from “IRAT129” could distribute more Cd to leaf sheaths than to leaf blades (*P* = 0.015; Figure 5C). Regarding Cd tolerance variation, only one minor QTL on chromosome 4 (*qCdtol4*) was identified, accounting for 5.43% of phenotypic variation with a positive effect of 0.068 (LOD = 2.69).

**TABLE 1:** Additive quantitative trait loci (QTLs) controlling variation in Cd distribution and Cd tolerance in the recombinant inbred line (RIL) population derived from “IRAT129” and “93-11.”

Indicators	QTLs	Chr.	Interval	LOD	Var^*a*^ (%)	Add^*b*^
Cd-Tol	*qCdtol4*	4	R4ID1855–R4ID2141	2.69	5.43	0.068
S/R	*qCddis4*	4	R4ID2816–R4ID3340	3.17	8.97	–0.041
	*qCddis8*	8	RM6215–R8ID2270	2.91	9.01	–0.041
Lf/Nd	*qCddis2*	2	R2ID2501–R2ID2668	3.22	6.22	–0.054
	*qCddis11*	11	RM21–R11ID2085	3.04	6.94	0.058
Lb/Ls	*qCddis1*	1	R1ID3282–R1ID4024	7.80	5.22	0.016
	*qCddis3.1*	3	R3ID1548–R3ID1658	4.19	2.56	–0.012
	*qCddis3.2*	3	R3ID2239–R3ID2458	6.90	4.60	0.016
	*qCddis3.3*	3	RM7389–RM85	3.30	1.99	–0.010
	*qCddis5*	5	R5ID1931–RM5329	4.19	2.69	–0.012
	*qCddis7.1*	7	R7ID0415–RM6081	8.96	6.08	0.018
	*qCddis7.2*	7	R7ID0903–RM3635	11.70	8.62	–0.021
	*qCddis7.3*	7	R7ID1506–R7ID1740	6.15	4.19	0.015
	*qCddis7.4*	7	R7ID2122–R7ID2850	3.89	2.52	–0.011
	*qCddis8*	8	RM6215–R8ID2270	9.76	7.20	–0.019
	*qCddis9*	9	RM201–R9ID2281	4.65	2.97	0.012
	*qCddis11*	11	R11ID0407–R11ID0542	2.75	1.77	0.010
	*qCddis12.1*	12	R12ID0014–R12ID0317	2.66	2.98	0.013
	*qCddis12.2*	12	R12ID1072–R12ID2189	3.24	2.00	–0.010

*Positive values imply positive effects from “93-11” alleles; negative values imply positive effects from “IRAT129” alleles. The Cd concentration ratio between shoots (leaves and nodes) to roots is represented as S/R, the Cd concentration ratio between developing tissues (leaf sheaths and leaf blades) and nodes is represented as Lf/Nd, and the Cd concentration ratio between leaf blades and leaf sheaths is represented as Lb/Ls.*

*^*a*^Phenotypic variation explained by the QTLs.*

*^*b*^Additive effects.*

A total of 24 epistatic loci were identified for explaining the variation in Cd tolerance, and one epistatic locus was identified for explaining the variation in S/R for Cd distribution (Table 2 and Figures 4C,D). Epistatic QTLs were detected on all 12 chromosomes, and interactions between different loci jointly contributed to the variation in Cd tolerance. Among the 24 epistatic loci for Cd tolerance variation, six loci were found to independently interact with multiple loci, located on chromosomes 1, 2, 4, and 5 (Table 2), implying hotspots of epistasis. Regarding Cd distribution variation due to epistatic effects in the population, one interaction between two loci on chromosome 2 was found to contribute to the phenotypic variation in S/R. The QTL analysis revealed that different genetic patterns drive Cd distribution and Cd tolerance variation in the RILs.

**TABLE 2:** Epistatic quantitative trait loci (QTLs) controlling variation in Cd distribution and Cd tolerance in the recombinant inbred line (RIL) population.

Trait	Interval of QTLA	Chr. of QTL1	Interval of QTL2	Chr. of QTL2	LOD	Add1 of QTL1^*a*^	Add2 of QTL2^*b*^	Add1 by Add2^*c*^
Cd-Tol	R1ID0401–R1ID0548	1	R1ID0548–RM1195	1	10.74	–0.329	0.336	–0.284
	R1ID4105–R1ID4255	1	R2ID3156–R2ID3326	2	8.69	–0.319	0.306	–0.302
			R3ID1077–R3ID1384	3	7.66	–0.322	0.304	–0.303
			R5ID0220–R5ID1614	5	9.26	–0.321	0.299	–0.312
	R2ID0562–R2ID0941	2	R2ID0941–R2ID1750	2	12.61	0.25	–0.242	–0.382
	R2ID2501–R2ID2668	2	R5ID1614–R5ID1931	5	10.30	–0.345	0.308	–0.324
	R2ID3156–R2ID3326	2	R3ID0572–R3M10	3	6.94	0.307	–0.303	–0.320
			R4ID2356–R4ID2816	4	10.08	0.312	–0.355	–0.309
			R6ID0227–R6ID0456	6	7.58	0.306	–0.31	–0.316
			RM346–RM6835	7	6.87	0.307	–0.312	–0.312
			R9ID0221–RMB23867	9	7.20	0.309	0.307	0.315
	R3ID1487–R3ID1548	3	R3ID1658–R3ID1848	3	11.58	0.312	–0.309	–0.309
	R3ID0572–R3M10	3	R5ID1614–R5ID1931	5	7.92	–0.322	0.306	–0.306
	R4ID2141–R4ID2356	4	R4ID2356–R4ID2816	4	12.85	–0.279	0.275	–0.410
	R5ID0220–R5ID1614	5	R5ID1614–R5ID1931	5	13.10	0.253	–0.256	–0.375
			R6ID1694–RM20242	6	9.61	0.302	–0.313	–0.317
			R8ID0023–R8ID0216	8	8.49	0.301	–0.311	–0.322
	RM20242–R6ID2116	6	R6ID2116–R6ID2316	6	11.71	–0.28	0.262	–0.354
	R7ID1506–R7ID1740	7	R7ID1740–RM346	7	10.67	–0.307	0.311	–0.312
	R8ID0023–R8ID0216	8	R8ID0216–RM72	8	11.17	–0.306	0.312	–0.312
	R9ID1738–OSR28	9	RM201–R9ID2281	9	11.00	–0.274	0.273	−−0.35
	R10ID0153–R10ID0741	10	R10ID0153–R10ID0741	10	8.34	0.318	–0.318	–0.300
	RM21–R11ID2085	11	R11ID2085–R11ID2170	11	11.90	–0.254	0.277	–0.361
	R12ID2189–RM277	12	RM277–R12ID2339	12	11.29	–0.316	0.316	–0.303
S/R	R2ID2035–RM1385	2	R2ID3077–R2ID3156	2	5.31	–0.07	0.068	–0.092

*Positive values represent the positive effect from “93-11” alleles; negative values represent the positive effect from “IRAT129” alleles. S/R represents the Cd concentration ratio between shoots (leaf sheaths, leaf blades, and nodes) to roots; Cd-Tol represents Cd tolerance.^*a,b*^Additive effects of QTL1 and QTL2.^*c*^Interaction effects for epistatic loci from additive loci between QTL1 and QTL2.*

### Additive Effects From Multiple Loci Determine Variation in Cd Distribution

In the present study, QTL analyses revealed that additive effects from loci, rather than interactions between loci, dominated the variation in Cd distribution in the RILs (Table 1). Thus, it could be assumed that Cd distribution phenotypes may be gradually enhanced along with increasing additive loci from the same allelic pools. Based on the results of QTL analyses, seven QTLs were found to exert negative effects on S/R, Lf/Nd, and Lb/Ls, which indicates that alleles from “IRAT129” could reduce the related phenotypic variation (Table 1). Specifically, in both of the 2 QTLs for S/R variation, 1 of the 2 QTLs for Lf/Nd variation, and 7 of the 14 QTLs for Ld/Ls variation, alleles from “IRAT129” decreased the phenotypic performance. According to their genotypes, we selected RIL line harboring different combinations of “IRAT129” alleles to investigate the variation in S/R and Lb/Ls. Using linear regression, we simulated the relationship between phenotypic variation in the population and the number of additive loci exerting negative effects on phenotypes. For variation in S/R and Lb/Ls, a significant decrease in phenotype was observed along with an increase in these loci (*P* < 0.05; Figures 5D,E). Correlation coefficients (*r*) between the number of additive loci and the phenotypes were 0.26 for S/R and 0.23 in Lb/Ls (*P* = 0.006, 0.019). Therefore, the results revealed that the additive effects from these loci determined the variation in Cd distribution in the RIL population.

Together, the results of QTL analysis revealed quite different genetic patterns between Cd distribution and Cd tolerance. Additive effects from loci played crucial roles in determining variation in Cd distribution, while interaction effects between different loci contributed to variation in Cd tolerance.

## Discussion

Extensive studies on Cd distribution and Cd tolerance in rice in recent decades has revealed details of the mechanisms underpinning these physiological processes (Clemens, 2006; Yamaji and Ma, 2014). As a result, a series of genes and QTLs associated with Cd distribution and Cd tolerance have been well established (Clemens et al., 2013). These studies demonstrate that Cd is translocated into different parts of rice plants through the Cd distribution system, including uptake, sequestration, transport, compartmentalization, and accumulation, where it causes local and systemic toxicity. Thus, it is necessary to investigate the genetic relationships between Cd distribution and Cd tolerance and identify the potential QTLs responsible for simultaneous variation in these traits to facilitate genetic improvement by reducing Cd accumulation and enhancing Cd tolerance in rice varieties. There is genotypic diversity in Cd distribution and Cd tolerance in different rice varieties (Uraguchi and Fujiwara, 2012; Pinson et al., 2015; Tan Y. J. et al., 2020). QTL analysis is a powerful genetic approach for exploring genetic differences and identifying markers or loci associated with phenotypic variation in biparental genetic populations. Herein, by employing this approach, we assessed Cd distribution and Cd tolerance differences in a RIL population based on genetic diversity between *indica* and *japonica* rice.

Recent studies on natural variation in mineral nutrients and toxic elements in rice were implemented using normalization of phenotypic variation to enhance genetic factors (Wang et al., 2020; Tan Y. J. et al., 2020). In our current study, the fact that a faster growth in “IRAT129” could be observed than that in “93-11” could result in unexpected differences in objective traits such as Cd transportation ability during different growth phases (Clemens and Ma, 2016), which may affect the accuracy of phenotypic identification. Meanwhile, as typical quantitative traits, Cd distribution and Cd tolerance traits are controlled by multiple loci, and environmental factors have significant effects on trait performance (Xue et al., 2009; Zhang et al., 2014; Tan Y. J. et al., 2020). The influence of environmental factors on phenotypic differences can make it difficult to accurately identify differences and stable genetic controls. Thus, in our study, we normalized phenotypic performance by developing three Cd distribution indicators and one Cd tolerance indicator to more reliably disclose potential genetic variation. Based on variation in the four phenotypic indicators, it was found out that Cd distribution differences between “IRAT129” and “93-11” were mainly due to Cd transportation from roots to nodes and from leaf sheaths to leaf blades (Figure 1C), and changes in growth status were much better for “IRAT129” than “93-11” under 25 μM CdSO_4_ treatment (Figure 2C). These indicators were also used to investigate genotypic variation in the RILs, and the results indicate that normalizing Cd-related traits could be widely used to easily and accurately determine allelic variation in QTL analysis.

Based on the normalization of Cd-related traits, a total of 18 additive QTLs and 1 locus were found to be associated with allelic variation in Cd distribution and Cd tolerance in the RIL population derived from “93-11” and “IRAT129” (Table 1 and Figure 4). Compared with previous studies using associated mapping and linkage mapping, except for detecting *qCdtol4* related to Cd tolerance for the first time, most of the additive QTLs for Cd distribution were mapped in the same genomic regions as QTLs reported previously (Sun et al., 2016; Hu et al., 2018; Chen et al., 2019; Liu X. et al., 2019; Liu W. et al., 2019; Tan Y. J. et al., 2020; Wang et al., 2020). There were still some differences present according to the results in our study. Among these QTLs, one pleiotropic locus, *qCddis8*, was found to simultaneously affect Cd distribution from roots to shoots, as well as Cd distribution from leaf sheaths to leaf blades (Table 1), and the *qCddis8* allele from “IRAT129” could reduce the phenotypic variation (Figures 5A,B). This pleiotropic locus has been repeatedly detected in many other studies, revealing great allelic diversity in this genomic region (Liu X. et al., 2019; Tan Y. J. et al., 2020; Wang et al., 2020). In addition, among the detected QTLs in our study (Table 1), QTL-*qCddis7.2* displaying the highest LOD value was detected on the short arm of chromosome 7, and this QTL affected Cd distribution from leaf sheaths to leaf blades (Table 1 and Figure 5C). In previous studies, a series of QTLs and genes on chromosome 7 were found to be associated with Cd accumulation (Ueno et al., 2010; Takahashi et al., 2011; Sasaki et al., 2012). To date, three Cd-related genes, *OsHMA3*, *OsNramp1*, and *OsNramp5*, have been found to control Cd uptake or sequestration, and these were located in the genomic region of *qCddis7.2* (Figure 4B). *OsNramp5* reportedly participates in Cd uptake in rice, and loss of function for this gene greatly reduces Cd uptake (Ishikawa et al., 2012). *OsNramp1* encodes a transporter responsible to Cd uptake and transport, and sequence variation in its promoter can generate variation in expression levels, which leads to differences in Cd levels in shoots (Takahashi et al., 2011). Allelic variation in *OsHMA3* has been confirmed to be responsible for variation in Cd accumulation in rice by sequestrating different amounts of Cd in root vacuoles (Miyadate et al., 2011; Sui et al., 2019; Liu C. et al., 2020). Thus, functional allele variation in these three genes may be the main genetic factor affecting Cd distribution via *qCddis7.2*. Thus, it is necessary to investigate functional allele variation in the related QTLs detected in this study, and the results could improve our genetic understanding of Cd distribution.

The results of QTL detections in our present study revealed two different genetic features controlling Cd distribution and Cd tolerance. First, there was a bias in the QTL working patterns between Cd tolerance and Cd distribution. Most of the loci (18 out of 19 QTLs) responsible for Cd distribution were identified as additive QTLs (Table 1 and Figures 4B,D), and most of the loci (24 out of 25 QTLs) for Cd tolerance were detected as epistatic loci (Table 2 and Figures 4A,C). Second, most of the detected QTLs, including additive QTLs and epistatic QTLs, did not share the same genomic regions simultaneously, except for the locus on chromosome 11 detected as an additive locus for Lf/Nd and an interacting locus for Cd-Tol (Tables 1, 2 and Figures 4B,C). These genetic features suggest that differences in Cd levels may not endow differences in Cd tolerance. On the other hand, there was a clear decrease in phenotypes with increasing loci responsible for S/R or Lb/Ls variation (Figures 5D,E), suggesting that additive effects from different loci drive phenotypic variation. Together, the above results revealed that quite different genetic patterns were responsible for variation in Cd distribution and Cd tolerance in our testing population; interaction effects between different loci led to variation in Cd tolerance, whereas additive effects from different loci drove variation in Cd distribution (Figures 4C,D).

At present, geneticists and plant breeders are trying to develop rice varieties that accumulate low levels of Cd and exhibit better Cd tolerance to ease the risk of excessive Cd consumption and yield losses due to Cd toxicity (Clemens et al., 2013; Zhao et al., 2015). The findings of the present study may help to facilitate genetic improvements for Cd distribution and Cd tolerance. Our results revealed that interaction effects and additive effects from different loci separately drive phenotypic variation in Cd tolerance and Cd distribution. Thus, pyramiding different additive loci could improve the Cd distribution performance, and this could be confirmed using different additive loci (Figures 5D,E). For Cd tolerance improvement, pyramiding might be more effective for developing Cd-tolerant varieties by selecting or mutating rice varieties rather than combining different loci, due to a lack of QTLs with additive effects.

Another important aspect of the present study was exploring loci to systematically reduce Cd transport in rice (Table 1). Although a few of rice varieties with lower grain-Cd accumulation have been successfully developed based on allelic variation in some QTLs or genes derived from *japonica* gene pools, such as QTLs on chromosome 7 (Lu et al., 2019; Zhou et al., 2019), few alleles have been identified and utilized in the genetic improvement of Cd levels in rice; hence, grain-Cd levels in improved rice varieties remain high in some regions (Zhao et al., 2015). Few alleles controlling Cd distribution differences have been identified, and systematic genetic improvements have not been carried out. Thus, effort should be made to identify the functional alleles governing variation in Cd during physiological processes. In the present study, various QTLs responsible for Cd distribution in rice were systematically identified (Table 1 and Figure 4B). After exploring their genetic effects and molecular roles in determining Cd distribution in rice, functional alleles and linked markers could be used as molecular tools to develop Cd-free rice varieties in future breeding programs. Based on the results of our QTL analysis, we propose different breeding strategies for genetic improvements in Cd distribution and Cd tolerance. QTL mapping identified a series of QTLs that may be applicable for systematically genetically improving Cd distribution and Cd accumulation in rice to develop low-Cd varieties in future breeding programs.

## Conclusion

In this present study, we performed a joint study of phenotypic investigations with QTL analysis to identify the genetic controls governing Cd distribution and Cd tolerance in a biparent population derived from *japonica* rice variety “IRAT129” and *indica* rice variety “93-11.” The results showed that the genetic patterns controlling Cd distribution and Cd tolerance are quite different. Additive effects from different loci contribute to the inheritance of Cd distribution. By contrast, epistatic effects from loci are the main factors determining Cd tolerance variation. Meanwhile, one pleiotropic locus, *qCddis8*, was found to simultaneously affect Cd distribution from both roots to shoots and from leaf sheaths to leaf blades.

## Data Availability Statement

The raw data supporting the conclusions of this article will be made available by the authors, without undue reservation.

## Author Contributions

LS and W-BT: methodology and supervision. Y-BC and J-RW: validation. Y-BC and Y-CC: formal analysis. LS and Y-CC: investigation. Y-BC and SL: data curation. Y-XZ and H-bD: writing—original draft preparation. LS: writing—review and editing. LS, W-BT, Y-XZ, and H-bD: visualization. All authors have read and agreed to the published version of the manuscript.

## Conflict of Interest

The authors declare that the research was conducted in the absence of any commercial or financial relationships that could be construed as a potential conflict of interest.

## Footnotes

1 http://shenghuan.shnu.edu.cn/ricemarker

2 http://www.gramene.org/
