# Supplementary material for: Genetic Control Diversity Drives Differences Between Cadmium Distribution and Tolerance in Rice
Source: Front Plant Sci. 2021 Feb 19;12:638095. doi: 10.3389/fpls.2021.638095 (PMC7933448; doi:10.3389/fpls.2021.638095)

**Legend of supplementary tables and figures**

**Supplementary table 1 Cd levels in tissues and four indicators performance for Cd distribution and Cd tolerance in the RIL population**

| Cd treatment | Phenotypes and Indicators for Cd distribution | Variation range | Average ± SD | CV% |
| --- | --- | --- | --- | --- |
| 2.5 μM | Root | 309.54~1089.75 mg/kg | 690.97±140.23 mg/kg | 20.3 |
|  | Node | 163.96~375.13 mg/kg | 253.20±45.86 mg/kg | 13.1 |
|  | Sheath | 60.35~260.81 mg/kg | 150.90±35.07 mg/kg | 23.4 |
|  | Leaf | 8.80~63.82 mg/kg | 24.86±9.23 mg/kg | 37.1 |
|  | S/R | 0.38~1.18 | 0.64±0.12 | 18.6 |
|  | Lf/Nd | 0.37~1.01 | 0.72±0.18 | 25.4 |
|  | Lb/Ls | 0.07~0.32 | 0.15±0.05 | 29.6 |
| Cd treatment | Phenotypes and Indicators for Cd distribution | Variation range | Average ± SD | C.V.% |
| 0 μM | Shoot length | 4.75~12.78 cm | 8.78±1.49 cm | 17.0 |
|  | Root length | 4.8~12.53 cm | 8.54±1.42 cm | 16.6 |
| 25 μM | Shoot length | 14.2~40.28 cm | 28.67±5.58 cm | 19.5 |
|  | Root length | 13.3~34.13 cm | 23.74±4.03 cm | 17.0 |
|  | Cd-Tol | 0.61~2.33 | 0.88±0.20 | 22.5 |

**Supplementary Figure 1 the linkage map of the RIL population from ‘IRAT129’ and ’93-11’ rice varieties**


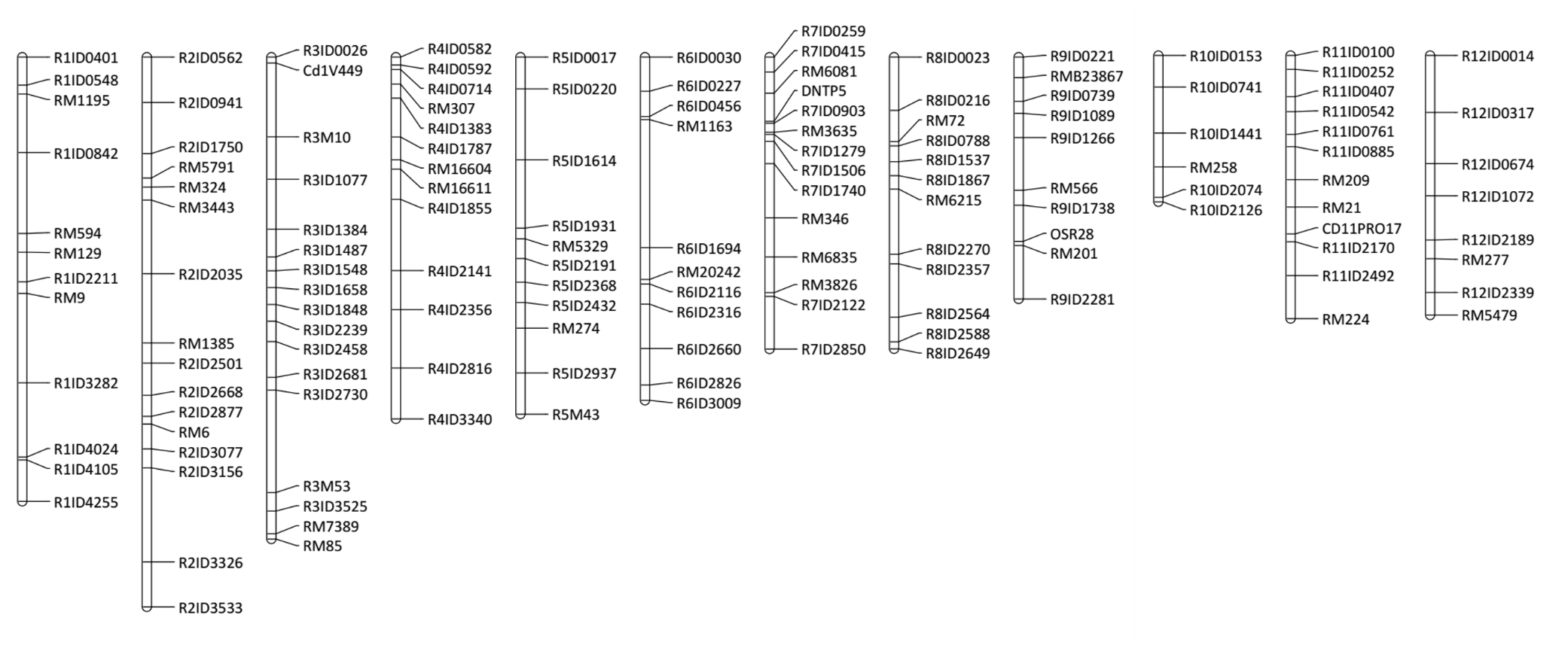

Supplement: Supplementary file 3 [file Data_Sheet_1.docx]
